# Simulation of microplastic transport and dispersion based on a three-dimensional hydrodynamic particle-tracking model in the Beibu Gulf

**DOI:** 10.3389/ftox.2025.1676823

**Published:** 2025-12-04

**Authors:** Changhao Sun, Yao Guan, Wenhao Hou, Huihua Wei, Xiaowei Hu, Huichang Jiang, Qiongyuan Su, Xiong Zhou, Jie Chen, Zuhao Zhu

**Affiliations:** 1 School of Resources, Environment and Materials, Guangxi University, Nanning, China; 2 Guangxi Key Laboratory of Beibu Gulf Marine Resources, Environment and Sustainable Development, Fourth Institute of Oceanography, Ministry of Natural Resources, Beihai, China; 3 Key Laboratory of Tropical Marine Ecosystem and Bioresource, Fourth Institute of Oceanography, Ministry of Natural Resources, Beihai, China; 4 Observation and Research Station of Coastal Wetland Ecosystem in Beibu Gulf, Ministry of Natural Resources, Beihai, China

**Keywords:** microplastics, Beibu Gulf, Lagrangian particle tracking, transport and dispersion, storm surge

## Abstract

The Beibu Gulf, a representative semi-enclosed bay in the South China Sea, experiences microplastic transport and dispersion governed by a complex interplay of monsoons, ocean circulation, and extreme weather events, warranting systematic investigation. We developed a numerical modeling framework by coupling a three-dimensional hydrodynamic model with a Lagrangian particle-tracking module, and validated it against in observations. The model quantitatively demonstrates high accuracy, with maximum spatial deviations below 6 km and relative standard deviations within 7%, confirming its suitability for simulating microplastic transport. The simulation results indicate that the transport of microplastics in the Beibu Gulf is primarily controlled by the oceanic hydrodynamic environment, while also being indirectly affected by the monsoon. During winter and autumn, the northeast monsoon dominates, whereas in spring and summer, the southwest monsoon prevails, with the overall circulation exhibiting a counterclockwise coastal current pattern. In spring, microplastics can disperse up to 205 km, while in summer, southwest monsoon conditions lead to the formation of nearshore high-concentration zones (∼20 μg/m^3^). Vertical transport significantly modulates plume structure, with summer pollution coverage expanding by over 70% compared to scenarios excluding vertical motion.Storm surge events further intensify hydrodynamic conditions. As a case study, Typhoon Yagi induced significant alterations in the hydrodynamic conditions of the Beibu Gulf: prior to the storm, tidal forces governed periodic flow variations; during and after the storm, intense circulations generated prominent counterclockwise vortices, with velocities reaching 2.8 m/s, substantially enhancing long-range microplastic transport and extending their spatial distribution. This study reveals the key characteristics of microplastic transport in the Beibu Gulf under varying seasonal and hydrodynamic conditions, providing a rigorous theoretical foundation for understanding regional microplastic dispersal patterns.

## 1 Introduction

With the increasing exploitation and utilization of marine resources by humans, various environmental issues have emerged in the ocean, among which plastic pollution has become one of the most prominent problems affecting marine ecosystems (Wang et al., 2018). A large amount of plastic waste enters the ocean from land through various pathways. It is estimated that plastics account for approximately 60%–80% of marine debris worldwide (Barnes et al., 2009; Auta et al., 2017; Thanigaivel et al., 2025). Microplastics (MPs) are typically defined as polymer particles smaller than 5 mm in size (Andrady, 2011), exhibiting diverse morphologies, including fibers, fragments, pellets, and films. They are generated from the gradual fragmentation of larger plastic debris in the marine environment under the influence of ultraviolet radiation, mechanical forces, and biodegradation processes (Cole et al., 2011). The predominant types of microplastics include polyethylene (PE), polypropylene (PP), and polystyrene (PS) (Gao et al., 2023). At present, microplastics are widely distributed in various environmental compartments, including surface seawater, deep-sea sediments, polar ice, and atmospheric deposition (Cózar et al., 2014; Peeken et al., 2018; Cunningham et al., 2020). It is estimated that the global abundance of plastic particles currently ranges from approximately 82–358 trillion pieces, with a total mass between 1.1 and 490 million tons (Eriksen et al., 2023). Marine microplastics have the capacity to adsorb a variety of organic pollutants (Koelmans et al., 2013), heavy metals (Li and Wang, 2023), and harmful microorganisms (Arias-Andres et al., 2019), posing potential risks to the marine ecological environment. Microplastics are readily ingested by marine organisms and can be transferred and accumulated through the food chain, posing a potential threat to human health (Carbery et al., 2018; Qiao et al., 2019). Approximately 75% of microplastics in the marine environment originate from land-based anthropogenic activities (Morales-Caselles et al., 2021) and are transported over long distances from coastal zones to the open ocean under the influence of oceanic and coastal currents.

Microplastic research primarily relies on *in situ* sampling, and the accuracy of observational data is highly dependent on the sampling process. Some observed results may be incidental or influenced by sampling variability (Liu et al., 2019). Moreover, long-term, real-time monitoring of microplastics at fixed locations remains challenging under current technological constraints (Yin et al., 2022). In recent years, the development and continuous refinement of ocean numerical models have provided an effective new approach for investigating oceanic physical processes, material transport, and pollutant dispersion. The primary modeling approaches for simulating microplastic transport include Eulerian models and the Lagrangian particle tracking models (Zhang, 2017). In the context of Eulerian approaches, Mountford and Morales Maqueda (2019) developed a three-dimensional model to reveal the vertical distribution of microplastics throughout the water column. Van Sebille et al. (2020) demonstrated that the interaction between Stokes drift and the Eulerian flow field significantly influences microplastic transport, and emphasized the need for future studies to couple wave and ocean models for more comprehensive investigation. In addition, backward particle tracking methods have been employed to trace the potential sources of microplastics (Sun et al., 2022). In the context of the Lagrangian particle tracking, Lebreton et al. (2012) coupled global ocean circulation with particle tracking to identify five major accumulation zones. Mansui et al. (2015) integrated the NEMO ocean model with a Lagrangian particle tracking approach to analyze the influence of Mediterranean circulation on microplastic transport. Iwasaki et al. (2017) employed particle tracking to reveal that microplastics in the Sea of Japan are transported northeastward under the influence of the Tsushima Current. Liu et al. (2023) utilized the Regional Ocean Modeling System (ROMS) model coupled with a Lagrangian transport model to investigate the seasonal and river discharge–driven characteristics of microplastic transport in Chinese coastal waters. The horizontal transport of microplastics is primarily driven by ocean circulation, wind forcing, and tides. Previous studies have indicated that ocean currents, winds, and river discharges facilitate the long-distance transport of microplastics (Zarfl and Matthies, 2010; Cole et al., 2011). Pan et al. (2019) revealed four distinct transport pathways under the combined influence of wind and current fields. In addition to hydrodynamic control, the vertical migration of microplastics is significantly affected by processes such as settling and resuspension. The particle density, shape, and size determine their distribution characteristics within the water column (Kumar et al., 2021); the sinking velocity is constrained not only by density and fluid properties but also by particle morphology (Kowalski et al., 2016). Long-term observations have shown that biofouling can alter particle density, leading to an increase in sinking rate over time (Karkanorachaki et al., 2021). Collectively, these processes govern the vertical migration pathways of microplastics in the water column. Reisser et al. (2015) observed an exponential decrease in microplastic abundance with depth, with most particles concentrated near the surface. Eriksen et al. (2014) suggested that microplastics eventually settle on the seafloor, while Kukulka et al. (2012) pointed out that wave-enhanced turbulence also influences their vertical distribution.

In recent years, research on microplastic pollution in China’s coastal waters has increased steadily (Zhao et al., 2014; Zhao et al., 2019; Sun et al., 2022; Chen et al., 2023; Gao et al., 2023; Ding et al., 2025). The Beibu Gulf, characterized by intensive coastal human activities, has experienced increasingly severe microplastic contamination (Zhu et al., 2023). Studies indicate that microplastics in the water of the Beibu Gulf are predominantly fragments, whereas fibers dominate in sediments, with higher abundances observed near urban areas. Microplastic concentrations are positively correlated with population density and economic development (Zhu et al., 2022). In seawater, microplastics are mainly white fibers, with terrestrial sources being dominant; the primary origins are household products and textiles, and nearshore waters exhibit higher concentrations than offshore regions (Zhu et al., 2023; Zhu et al., 2025). Sediments also contain widespread microplastics, whose distribution is jointly influenced by hydrodynamics, geological conditions, and anthropogenic activities, with the presence of highly toxic polymers further increasing ecological risks (Wu et al., 2025). The hydrodynamics of the Beibu Gulf are complex, with pronounced seasonal circulation patterns: cyclonic circulation dominates in spring; summer circulation exhibits cyclonic patterns in the north and anticyclonic patterns in the south; autumn and winter are characterized by a dual north-south cyclonic structure, with the cold-water mass forming in spring, peaking in summer, and dissipating in autumn (Gao et al., 2017). The primary water masses include the Coastal Current (CC), West Guangdong Coastal Current (WGCC), and South China Sea Water (SCSW), whose seasonal intrusions regulate salinity and nutrient distributions within the gulf (Lao et al., 2022). Although previous studies have investigated microplastic pollution and hydrodynamics in the Beibu Gulf, the coupling between microplastic distribution and hydrodynamic processes remains limited. The mechanisms governing microplastic dispersion, transport, and seasonal variability are still unclear. Moreover, the Beibu Gulf is frequently subjected to typhoons, with storm surges exerting a significant regulatory influence on regional material transport. Previous studies have demonstrated that typhoons disrupt thermal stratification and induce upwelling, thereby reshaping nutrient sources and distributions (Lao et al., 2023). The intense hydrodynamic disturbances triggered by storm surges are likely to further facilitate the migration and dispersion of microplastics, warranting further investigation through numerical modeling. In this study, a three-dimensional hydrodynamic model coupled with a Lagrangian particle-tracking module is developed to elucidate the spatial distribution patterns of microplastics in the Beibu Gulf and to further explore the roles of vertical transport and storm surges in governing their migration and dispersion.

## 2 Methods

### 2.1 Study area

**FIGURE 1:**
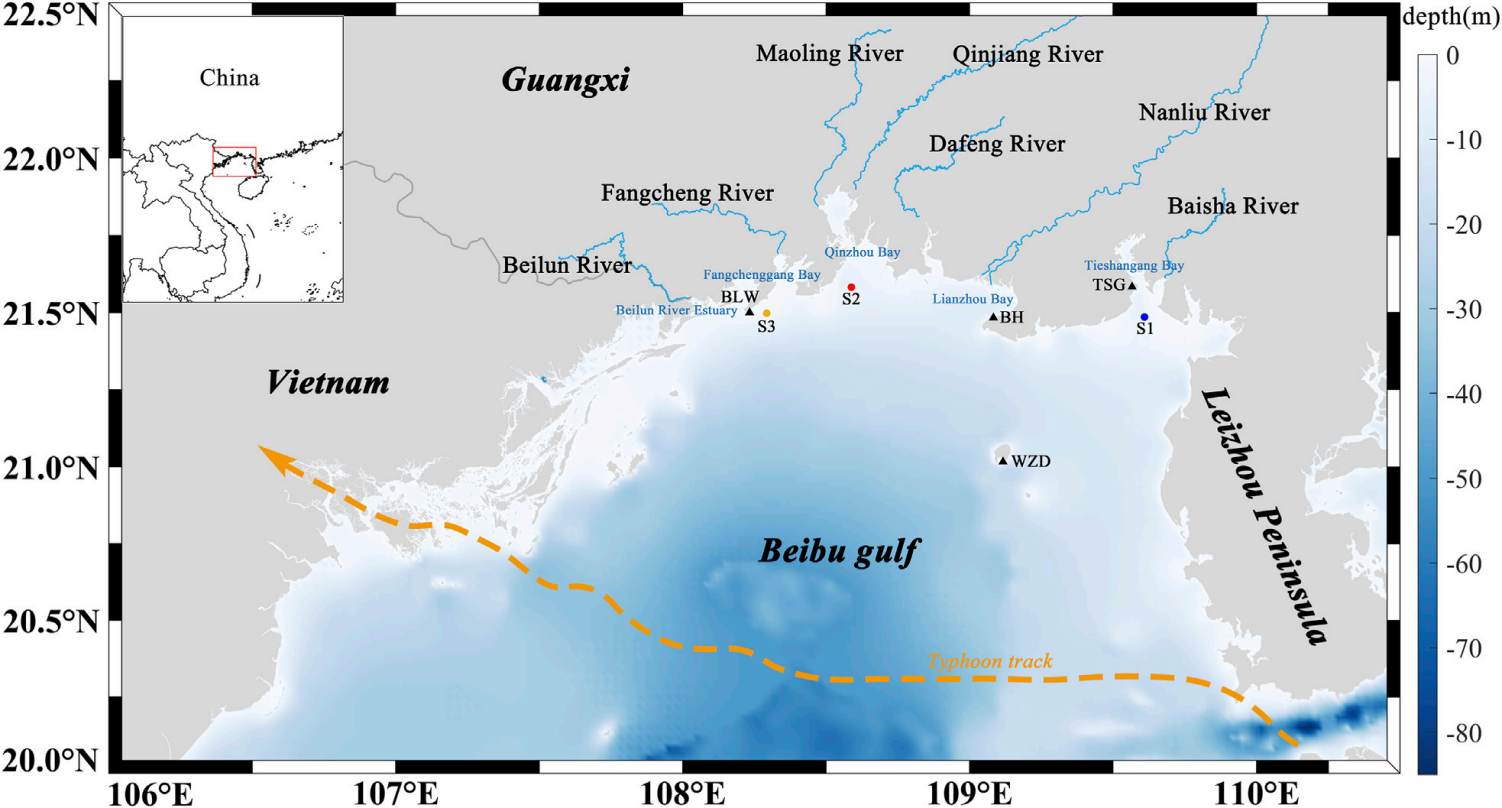
Study area and distribution of rivers and bays. The orange arrow indicates the track of Typhoon Yagi (international ID: 2411). Triangles denote tide gauge stations, and circles represent surface drifter release sites.

**FIGURE 2:**
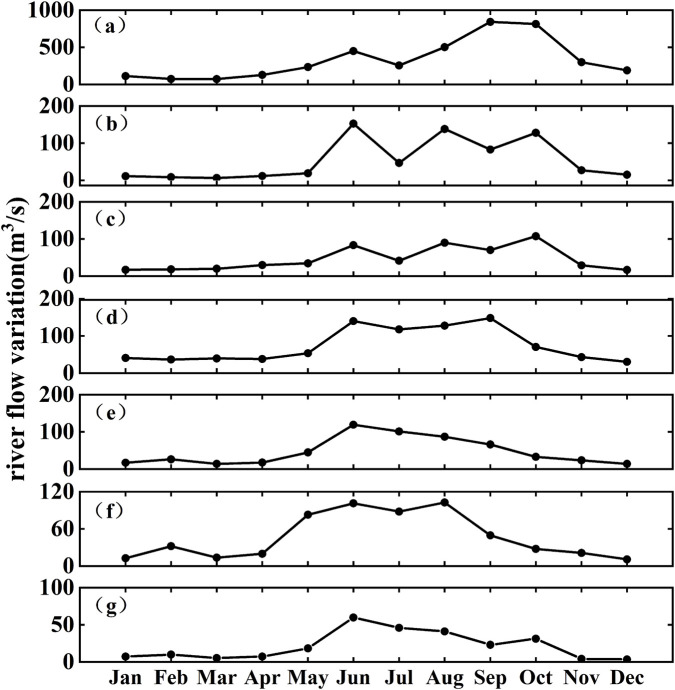
Monthly average river discharge time series in the Beibu gulf region in 2023. **(a)** Nanilu river. **(b)** Dafeng river. **(c)** Qinjiang river. **(d)** Maoling river. **(e)** Fangcheng river. **(f)** Beilun river. **(g)** Baisha river.

The Beibu Gulf is located in the northwestern part of the South China Sea and is a semi-enclosed bay bordered by land on three sides. The seafloor gradually deepens from the nearshore zones toward the central region, with an average depth of approximately 40 m and a maximum depth of less than 100 m. This region is characterized by a pronounced monsoonal climate, with prevailing southwesterly winds in summer and northeasterly winds in winter (Shen et al., 2018), and features persistent northward subtidal currents maintained by tidal-induced residual currents (Liu et al., 2025). This study focuses on the northern Beibu Gulf, where the study area encompasses five representative bays and seven major rivers that discharge into the sea (Figure 1). Rivers provide significant land-based inputs to coastal bays (Kanhai et al., 2022), and serve as critical conduits linking terrestrial and marine systems. They play a key role in transporting nutrients, organic matter, and microplastics, and represent the primary pathway through which microplastics are delivered from land to the ocean (Deng et al., 2025). River runoff in the Beibu Gulf exhibits pronounced seasonal variability (Figure 2). According to river discharge monitoring data provided by the Marine Environmental Monitoring Center Station, the Nanliu River has the highest annual average discharge among the seven major rivers entering the sea (Figure 2a). The Nanliu River enters its high-flow period between August and September, with the peak discharge occurring in September at approximately 840 m^3^/s. The low-flow period extends from January to March, with the lowest discharge observed in March. The other six rivers exhibit relatively lower discharge volumes and show similar intra-annual variation patterns, with their high-flow periods mainly concentrated between June and October.

### 2.2 Numerical model

#### 2.2.1 Governing equation

This study employs a three-dimensional hydrodynamic model coupled with a Lagrangian particle tracking module. The hydrodynamic component is based on the solution of the three-dimensional incompressible Reynolds-averaged Navier-Stokes equations, under the assumptions of Boussinesq approximation and hydrostatic balance. The model satisfies both the continuity and momentum equations, as shown in Equations 1–4:

$$\frac{\partial u}{\partial x}+\frac{\partial v}{\partial y}+\frac{\partial w}{\partial z}=0$$ (1)

$$\frac{\partial u}{\partial t}+\frac{\partial u^{2}}{\partial x}+\frac{\partial vu}{\partial y}+\frac{\partial wu}{\partial z}=fv-g\frac{\partial \eta }{\partial x}-\frac{1}{\rho_{0}}\frac{\partial p_{a}}{\partial x}+F_{u}+\frac{\partial }{\partial z}\left(\nu_{t}\frac{\partial u}{\partial z}\right)$$ (2)

$$\frac{\partial v}{\partial t}+\frac{\partial v^{2}}{\partial y}+\frac{\partial uv}{\partial x}+\frac{\partial wv}{\partial z}=-fu-g\frac{\partial \eta }{\partial y}-\frac{1}{\rho_{0}}\frac{\partial p_{a}}{\partial y}+F_{v}+\frac{\partial }{\partial z}\left(\nu_{t}\frac{\partial v}{\partial z}\right)$$ (3)

$$\frac{\partial p}{\partial z}=-\rho_{0}g$$ (4)

where *t* is time; *x*, *y*, *z* are Cartesian coordinates; *η* is the water surface elevation; *d* is the still water depth; *h = η + d* is the total water depth; *u, v, w* are the velocity components in the *x, y, z* directions, respectively; *f* is the Coriolis parameter; *g* is the gravitational acceleration; *ρ* is the water density; *v*_*t*_ is the vertical eddy viscosity coefficient; *P*_*a*_ is the atmospheric pressure; *ρ*_*0*_ is the reference water density and *F*_*u*_ *, F*_*v*_ denote the horizontal stress terms represented using stress gradients.

The fundamental principle of the particle-tracking model is to assume that particles are transported under the influence of natural forces such as water currents or wind, with diffusion represented by the addition of a stochastic random-walk term. The differential form of the governing equation is shown in Equation 5:

$$dX_{t}=a\left(t,X_{t}\right)dt+b\left(t,X_{t}\right)\xi_{t}dt$$ (5)

Where *X*_*t*_ represents the system state variable, such as particle position or concentration; *a* (t, *X*_*t*_) is the drift term, describing the mean rate of change of the system, with units of [*X*_*t*_]/[t]; *b* (t, *X*_*t*_) is the diffusion coefficient, controlling the magnitude of the stochastic perturbation; and *ξ*_*t*_ represents a random perturbation or noise, such as the random forces generated by molecular collisions in Brownian motion. When multiplied by *b* (t, *X*_*t*_) and applied over dt, *ξ*_*t*_ has the same units as *dX*_*t*_, together describing the evolution of the system under both deterministic drift and stochastic forcing.

#### 2.2.2 Numerical solution

The spatial discretization of the computational domain was performed using the Finite Volume Method, in which the continuous domain is subdivided into non-overlapping triangular elements as shown in Equation 6.

$$\frac{\partial U}{\partial t}+\nabla \cdot F\left(U\right)=S\left(U\right)$$ (6)

where U is the conserved physical variable vector, F is the flux vector, and S is the source term.

Spatial discretization was performed using a second-order scheme, with spatial accuracy enhanced through a linear gradient reconstruction technique. The gradients were computed using the weighted averaging method proposed by Jawahar and Kamath (2000). To suppress numerical oscillations inherent in high-order schemes, a second-order total variation diminishing (TVD) scheme was employed for flux limiting.

The time integration of the three-dimensional model was performed using a semi-implicit scheme, in which the horizontal terms were treated implicitly, while the vertical terms were handled using fully implicit, partially explicit, and partially implicit approaches. In general, a semi-implicit equation can be expressed as Equation 7:

$$\frac{\partial U}{\partial t}=G_{h}\left(U\right)+G_{v}\left(BU\right)=G_{h}\left(U\right)+G_{v}^{I}\left(U\right)+G_{v}^{V}\left(U\right)$$ (7)

In the equation, the subscripts *h* and *v* refer to the horizontal and vertical terms, respectively, while the superscripts *I* and *V* denote inviscid and viscous components.

We employed a high-order scheme for the three-dimensional transport equation, as shown in Equations 8 and 9:

$$U_{n+1/2}-\frac{1}{4}\Delta t\left(G_{V}^{V}\left(U_{n+1/2}\right)+G_{V}\left(U_{n}\right)\right)=U_{n}+\frac{1}{2}\Delta tG_{h}\left(U_{n}\right)+\frac{1}{2}\Delta tG_{V}^{I}\left(U_{n}\right)$$ (8)

$$U_{n+1/2}-\frac{1}{2}\Delta t\left(G_{V}^{V}\left(U_{n+1/2}\right)+G_{V}^{V}\left(U_{n}\right)\right)=U_{n}+\Delta tG_{h}\left(U_{n+1/2}\right)+\Delta tG_{V}^{I}\left(U_{n+1/2}\right)$$ (9)

The horizontal and vertical advection terms were integrated using a second-order Runge-Kutta scheme, while the vertical term was integrated using a second-order implicit trapezoidal rule.

The boundary conditions include the following: at land or solid boundaries, the normal velocity is set to zero to prevent fluid penetration; at open boundaries, time-varying water levels or discharge are imposed to represent tidal or runoff forcing; in areas with alternating wet and dry conditions, a dynamic wetting–drying algorithm proposed by Zhao et al. (1994) and Sleigh et al. (1998) is applied. In this approach, cells are classified as dry, partially wet, or wet based on local water depth h, with corresponding flux calculation rules applied, ensuring that *h*_*dry*_ < *h*_*flood*_ < *h*_*wet*_. Cells with water depth below the dry threshold are excluded from computations, while in shallow or transitional areas, only the continuity equation is solved.

#### 2.2.3 Model configuration

The model was configured with a time step of 180 s. The horizontal computational domain was discretized using an unstructured triangular grid, and the vertical direction was divided into three layers after applying the σ-coordinate transformation (Haidvogel et al., 1991). A total of 197,859 grid cells were generated within the computational domain, with spatial resolution gradually transitioning from 60 m near the coastline to 5,000 m at the open boundary, balancing the need for nearshore detail and large-scale dynamic representation. To systematically investigate the seasonal dispersion characteristics of microplastics, the model was independently simulated for each of the four seasons. The initial condition was set to zero, and each simulation was run for 90 days, including a 24-h spin-up time to ensure the stability of the model dynamics. The spatial distribution of microplastics for each season was extracted at the end of the simulation period. In the model, rivers were represented as point sources, while no microplastic flux was prescribed at the open ocean boundaries. The model was driven by tidal forcing conditions. The tidal elevations at each grid node along the open boundary were obtained from the MIKE Global Tide Model, which determines the harmonic constants based on 8 major tidal constituents (M2, S2, N2, K2, K1, O1, Q1 and P1). The tidal elevation is calculated as Equation 10:

$$\eta =\sum_{i=1}^{8}f_{i}h_{i}\cos\left(\omega_{i}t+v_{0i}+u_{i}-g_{i}\right)$$ (10)

Where *η* represents the predicted tidal elevation at the open boundary; *f*_*i*_ is the nodal factor of the *i*th tidal constituent; *h*_*i*_ and *g*_*i*_ denote the amplitude and phase lag of the *i*th constituent, respectively; *ω*_*i*_ is the angular frequency of the *i*th constituent; and *v*_*0i*_ + *u*_*i*_ represents its astronomical argument.

Bathymetric data were obtained from NOAA (https://www.ngdc.noaa.gov/) and supplemented with local nautical charts. Atmospheric forcing was derived from ERA5 reanalysis data provided by ECMWF (https://www.ecmwf.int/), with hourly 10 m wind speed and mean sea level pressure from a single vertical level extracted as inputs. In the particle tracking module, this study parameterized the degradation, settling, and resuspension processes of microplastics (Table 1). Observational data were obtained during two research cruises in the Beibu Gulf in 2020, namely, “Yueke 1” and “Yuexiayuzhi 20028,” yielding a total of 75 seawater samples and 66 surface sediment samples. Seawater samples were collected using a Manta trawl equipped with a 330 μm nylon mesh, while surface sediment samples (sampling depth >5 cm) were obtained with a Van Veen grab sampler. In the model, the microplastic input concentrations from riverine point sources were set based on measurements from sampling sites near the estuaries. Based on field measurements, this study selected the dominant type of microplastic in the Beibu Gulf—polystyrene (PS) fragments for analysis. The fluxes of microplastic inputs were estimated using observed microplastic abundances, particle volume and density, and river discharge (Table 2). The model outputs are presented in terms of concentration (µg/m^3^). For the storm surge simulations, the 2024 super typhoon “Yagi” (formation: 1 September 2024; dissipation: 8 September 2024) was selected as the study case, with relevant data obtained from the China Meteorological Administration Typhoon Network (https://typhoon.nmc.cn/web.html). Based on this typhoon event, the numerical model was employed to investigate the mechanisms by which storm-induced hydrodynamic processes influence microplastic dispersion and transport.

**TABLE 1:** Model parameter setting.

Process	Unit	Parameter	References
Decay	% per day	0.01	Andrady (2011)
Settling	m/s	0.004	Karkanorachaki et al. (2021)
Erosion	N/m^2^	0.01	Zhao et al. (2024)

**TABLE 2:** The riverine input flux of microplastics (unit: mg/s).

River	Spring	Summer	Autumn	Winter
Beilun River	11.96	29.96	10.11	5.73
Fangcheng River	7.54	30.01	12.02	5.66
Maoling River	0.94	3.01	1.59	0.89
Qinjiang River	1.24	3.13	3.02	0.77
Dafeng River	0.60	5.38	3.80	0.57
Nanliu River	5.22	14.42	23.33	4.54
Baisha River	0.57	2.68	1.07	0.38

### 2.3 Model validation data

Tidal level data were validated using observations from four tide gauge stations located within the Beibu Gulf (Figure 1). In addition, to verify particle transport trajectories, three surface drifters were deployed in the Beibu Gulf in June 2025 to collect in surface current and transport path data for comparison with the model results (Table 3). Each drifter consisted of a float, a positioning system, and a control mechanism, and was equipped with a real-time positioning module supporting both GPS and BeiDou satellite systems. The floating depth was adjusted by regulating the internal water volume to ensure that the drifters remained in the surface layer of the water column. Drifter trajectories were recorded at 3-min intervals, providing validation data for the simulation of microplastic transport.

**TABLE 3:** Surface drifter deployment information.

Label	Release location			Release time (UTC)	
	Bay	Lon	Lat	Start time	End time
S1	Tieshangang Bay	109.61°	21.49°	Jun.6 12:06,2025	Jun.6 23:27,2025
S2	Qinzhou Bay	108.59°	21.58°	Jun.11 10:32,2025	Jun.12 08:20,2025
S3	Fangchenggang Bay	108.29°	21.50°	Jun.11 08:53,2025	Jun.12 11:26,2025

### 2.4 Assessment of Lagrangian particle tracking performance

In this study, the performance of the Lagrangian particle tracking model was evaluated using the correlation coefficient (R), mean absolute error (MAE), root mean square error (RMSE), and relative separation distance (RSD) (Hu et al., 2023; Kang and Xia, 2025). The correlation coefficient (R) is used to measure the linear relationship between the simulated and observed values, reflecting the model’s ability to capture temporal trends. The mean absolute error (MAE) characterizes the average magnitude of absolute deviations between simulated and observed values, providing an intuitive assessment of overall error. The root mean square error (RMSE), which places greater emphasis on larger deviations, quantifies the overall level of discrepancy. The relative separation distance (RSD) evaluates the spatial agreement between simulated and observed trajectories, serving as an indicator of the model’s capability to reproduce particle spatial distribution as shown in Equations 11–14.

$$R=\frac{\sum_{i=1}^{n}\left(o_{i}-\bar{o}\right)\left(m_{i}-\bar{m}\right)}{\sqrt{\sum_{i=1}^{n}{\left(o_{i}-\bar{o}\right)}^{2}}\cdot \sqrt{\sum_{i=1}^{n}{\left(m_{i}-\bar{m}\right)}^{2}}}$$ (11)

$$MAE=\frac{1}{n}\sum_{i=1}^{n}\left(o_{i}-m_{i}\right)$$ (12)

$$RMSE=\sqrt{\sum_{i=1}^{n}{\left(o_{i}-\bar{o}\right)}^{2}/n}$$ (13)

$$RSD=\frac{d_{n}}{\sum_{i=1}^{n}l_{oi}}$$ (14)

Where *o* represents the observed data, *m* denotes the model output, *d*_*n*_ is the positional distance between the modeled particle and the drifter, and *l*_*oi*_ is the total displacement of the drifter.

## 3 Results and discussion

### 3.1 Comparison between drifter trajectories and simulated particle tracking paths

In June 2025, three surface drifters were deployed in the Beibu Gulf to obtain nearshore drift characteristics. The first drifter (ID: S1, blue in Figure 1) was released in Tieshan Bay at 12:06 UTC on June 6, and was tracked for approximately 13 h. Due to grounding in shallow waters, the usable data were limited. The total drift distance was 5.43 km, with a general northwestward trajectory. The drifter exhibited oscillatory movement under tidal forcing, with a maximum and mean drift velocity of 0.49 m/s and 0.14 m/s, respectively. The second drifter (ID: S2, red in Figure 1) was deployed in the outer Qinzhou Bay at 10:32 UTC on June 11, and was tracked for approximately 1 day. It drifted predominantly from northeast to southwest, covering a total distance of 21.82 km, with maximum and average velocities of 0.67 m/s and 0.28 m/s, respectively. The third drifter (ID: S3, orange in Figure 1) was deployed in Fangcheng Bay at 08:53 UTC on June 11, with a tracking duration of about 1 day. Its drift path also followed a northeast-to-southwest direction, with a total displacement of 19.12 km. The maximum and mean drift speeds were 0.44 m/s and 0.26 m/s, respectively.

**FIGURE 3:**
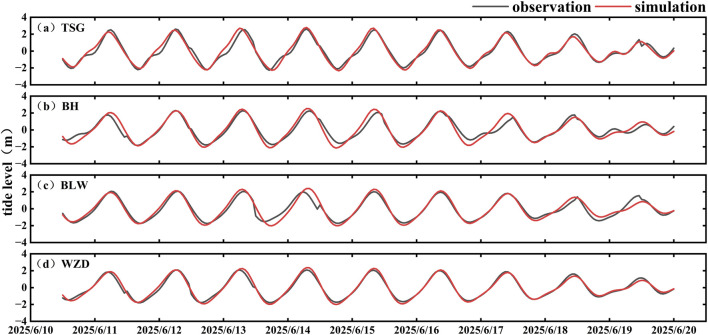
Tidal data validation. **(a)** Located at Tieshangang; **(b)** Located at Beihai; **(c)** Located at Fangchenggang; **(d)** Located at Weizhou Island.

**FIGURE 4:**
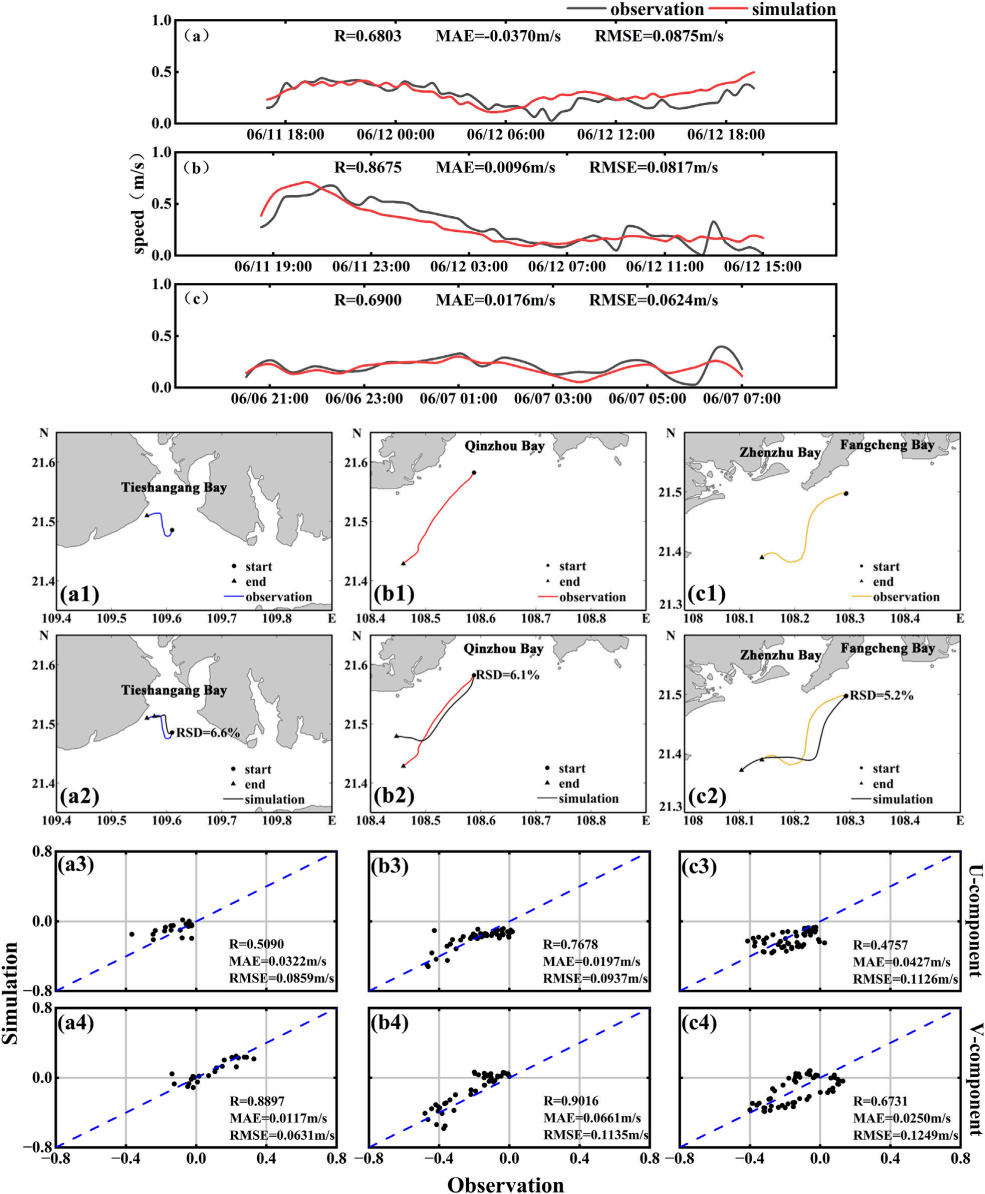
**(a–c)** Comparisons between observed and simulated velocities for surface drifters S1-S3. For each drifter: (a1-a4) correspond to S1; (b1-b4) to S2; and (c1-c4) to S3. The subplots show the observed trajectory, trajectory comparison, and scatter plots of velocity components.

To evaluate the reliability of the particle tracking model, we first validated the simulated tidal levels against observations from four tide gauge stations (Figure 3). Subsequently, the modeled particle drift trajectories were compared with the observed paths of the three surface drifters (Figure 4). For drifter S1, the simulated drift speed showed good correlation with the observed values, with an R value of 0.6900, MAE of 0.0176 m/s, and RMSE of 0.0624 m/s (Figure 4a). The correlation coefficients for the u and v-components were 0.5090 and 0.8897, with corresponding MAE values of 0.0322 m/s and 0.0117 m/s, and RMSE values of 0.0859 m/s and 0.0631 m/s (Figures 4a3, 4a4). The maximum spatial deviation was 1.43 km, and the relative separation distance (RSD) was 6.6% (Figure 4a2). For drifter S2, the modeled drift speed was well reproduced, with R = 0.8675, MAE = 0.0096 m/s, and RMSE = 0.0817 m/s (Figure 4b). The u and v-component R values were 0.7678 and 0.9016, with MAE values of 0.0197 m/s and 0.0661 m/s, and RMSE values of 0.09387 m/s and 0.1135 m/s (Figures 4b3, 4b4). The simulated trajectory closely matched the observed path during the initial phase, but the deviation increased during the flood tide due to localized hydrodynamic influences. The maximum spatial deviation reached 5.70 km, with an RSD of 6.1% (Figure 4b2). For drifter S3, the simulation also demonstrated strong agreement with the observations. The correlation coefficient for drift speed was 0.6803, with MAE and RMSE values of 0.0370 m/s and 0.0875 m/s, respectively (Figure 4c). The R values for the u and v-components were 0.4757 and 0.6731, with MAE values of 0.0427 m/s and 0.0250 m/s, and RMSE values of 0.1126 m/s and 0.1249 m/s (Figures 4c3, 4c4). In the Fangchenggang Bay region, the simulated trajectory exhibited overall good agreement with the observed path, with a maximum spatial deviation of 4.31 km and an RSD of 5.2% (Figure 4c2).

As Lagrangian particle tracking is highly sensitive to initial conditions and boundary forcing, and particle trajectories respond strongly to environmental perturbations, many studies have highlighted the challenges associated with its validation (Havens et al., 2010; Wei et al., 2016; Khatmullina and Chubarenko, 2019). Given the highly dynamic and complex nature of the marine environment, accurately reproducing the trajectory of an individual drifter remains challenging. The particle-tracking model employed in this study, which explicitly accounts for key hydrodynamic mechanisms such as tidal variability and wind-induced forcing, demonstrates reasonable skill in replicating drift velocities, directions, and trajectories, thereby exhibiting a credible level of reliability. However, the surface drifters used in this study were constrained by short tracking durations, preventing full coverage of all stages of microplastic transport. This limitation restricts the ability to comprehensively evaluate long-term transport pathways and the cumulative effects of external disturbances, potentially introducing uncertainty when characterizing the prolonged migration and accumulation of microplastics. In addition, temporal mismatches between the simulation period and the corresponding observational year may lead to discrepancies between model results and measurements. To enhance the applicability and reliability of the model for large-scale and long-term processes, future studies should consider incorporating longer time series of drifter observations or integrating other long-term monitoring data to achieve greater consistency in external forcing conditions, thereby improving the robustness and reliability of the simulation results. Despite these limitations, comparative analyses between model simulations and observed drifter trajectories demonstrate that the model achieves a high degree of accuracy in reproducing regional particle transport patterns. These results provide both robust technical support and a theoretical foundation for advancing the understanding and prediction of drift dynamics in the Beibu Gulf.

### 3.2 Seasonal characteristics of microplastic distribution

**FIGURE 5:**
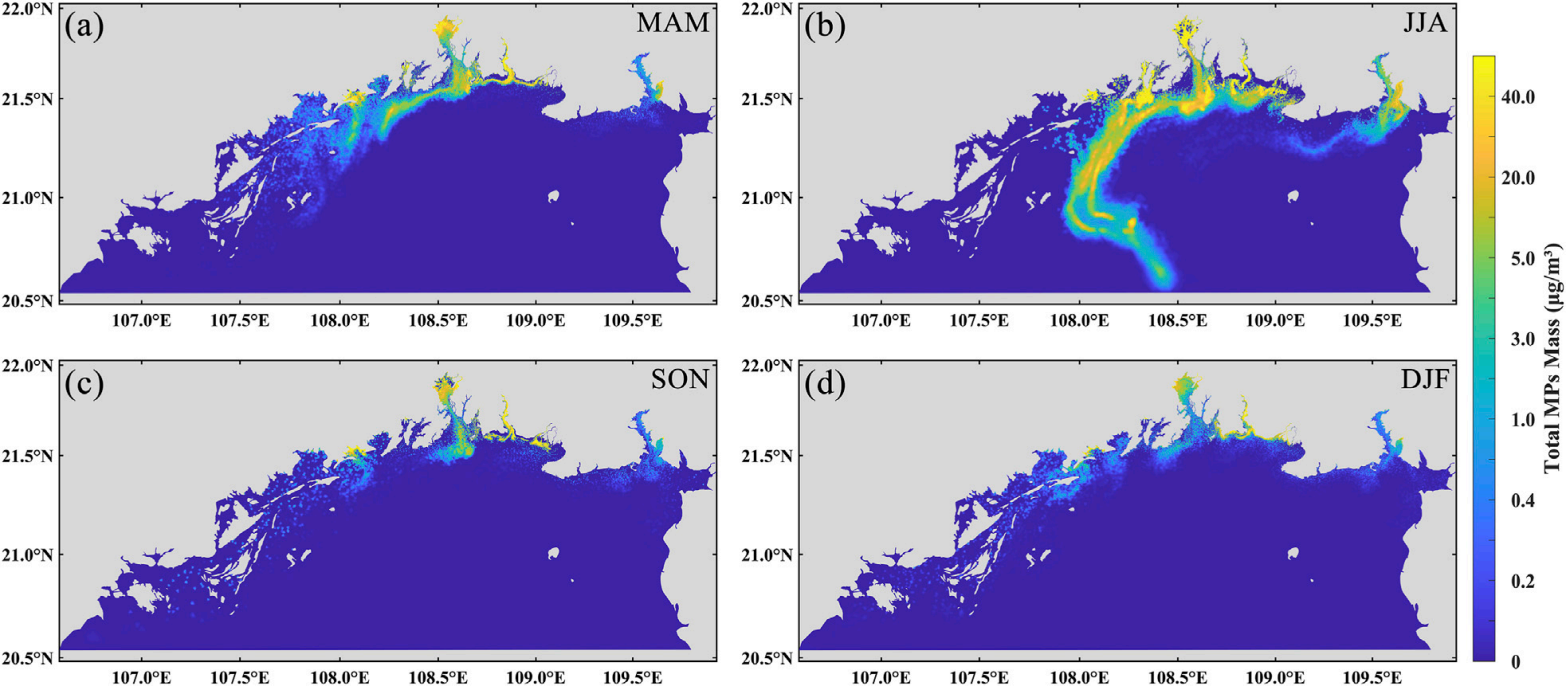
Spatial distribution of microplastics by season. **(a)** Spring MAM (March-May); **(b)** Summer JJA (June-August); **(c)** Autumn SON (September-November); **(d)** Winter DJF (December-February).

The maximum dispersion distance of microplastic pollution plumes increases over time in all seasons, but exhibits significant seasonal variation (Figure 5). In spring, autumn, and winter, the transport patterns are relatively similar, with microplastics predominantly dispersing southwestward along the coast. In spring, microplastics enter the gulf from river estuaries, with part of the particles continuing westward along the western shoreline, while others spread toward the central and northeastern parts of the gulf (Figure 5a). In autumn, the transport pathway shifts back to a primarily southwestward direction (Figure 5c). During winter, under the influence of prevailing northeasterly monsoons, with microplastics transported westward along the Chinese coast and then southward along the Vietnamese coast (Figure 5d). In contrast, summer is characterized by the influence of southwesterly monsoons, which drive microplastics offshore and northward, resulting in an offshore transport path toward the central Beibu Gulf (Figure 5b). The maximum transport distance in winter is approximately 180 km—the shortest among all four seasons. Autumn shows a similar extent of transport, with a maximum dispersion distance of around 170 km. In comparison, spring exhibits faster and broader microplastic dispersion, with particles reaching up to approximately 205 km from the source. In summer, although the maximum distance (∼185 km) is shorter than that in spring, high-concentration zones are rapidly carried offshore over a short time span. Both winter and autumn are dominated by northeasterly monsoons. Microplastics are transported southwestward along the Guangxi coastline and gradually accumulate along the Vietnamese coast. High-concentration zones are primarily located from the estuaries toward the southwestern part of the gulf and along the Vietnam shoreline, while low-concentration areas extend into the southwestern open sea. In contrast, spring and summer are influenced by southwesterly monsoons. Unlike the nearshore-dominated transport observed in winter and autumn, microplastic movement in these seasons is primarily directed offshore and toward the central gulf from the river mouths. The areal extent of high-concentration microplastic pollution zones varies significantly by season. The largest extent occurs in summer, covering approximately 4,968 km^2^ followed by spring at around 2,666 km^2^ (Table 4). Autumn and winter exhibit smaller affected areas, approximately 1,278 km^2^ and 2,006 km^2^ (Table 4), respectively. Distinct seasonal differences in microplastic concentrations are also observed between nearshore (0–20 km) and offshore (>20 km) zones. Concentrations are highest near riverine sources and lowest in open waters. In winter, spring, and autumn, nearshore concentrations exceed 40 μg/m^3^, whereas offshore values are mostly below 1 μg/m^3^, with some regions approaching background levels (<0.1 μg/m^3^), indicating that pollution plumes are largely confined to coastal zones. In summer, however, in addition to elevated nearshore concentrations, a prominent high-concentration region (∼20 μg/m^3^) appears between 20 km and 80 km offshore—significantly higher than in other seasons. This offshore enrichment is unique to summer and suggests that microplastics are effectively transported into the open sea. In contrast, offshore concentrations in winter, spring, and autumn remain consistently low, with no evidence of similar accumulation.

**TABLE 4:** Temporal evolution of microplastic dispersion area in each season (unit: km^2^).

Season	15 days	30 days	45 days	60 days	75 days	90 days
Spring (MAM)	105	246	411	927	1722	2666
Summer (JJA)	412	827	2431	3,149	4,353	4,968
Autumn (SON)	234	307	455	621	854	1,278
Winter (DJF)	415	743	992	1,432	1767	2006

The results indicate pronounced seasonal variability in microplastic dispersion within the Beibu Gulf. During winter and autumn, under the influence of the northeast monsoon, microplastics predominantly migrate southwestward along the coast, forming distinct accumulation zones along the Vietnamese shoreline, with a relatively limited dispersal range. In contrast, during spring and summer, when the southwest monsoon prevails, microplastics more readily disperse toward offshore areas and the central gulf, with summer exhibiting mesoscale high-concentration enrichment extending from nearshore to mid-shelf regions. These results indicate that the transport of microplastics in the Beibu Gulf is primarily regulated by ocean circulation, while the monsoon indirectly influences microplastic transport by altering the dynamics of the upper-layer seawater. Analysis of the dispersion maps shows that the overall hydrodynamic characteristics are consistent across the four seasons, with coastal currents exhibiting a predominantly counterclockwise pattern, aligning well with previous hydrodynamic studies in the Beibu Gulf (Gao et al., 2017). Similarly, Wang et al. (2018) reported a summer transport tendency of coastal particles toward the central gulf. Prior studies further indicate that microplastic migration and dispersion in marine environments are jointly controlled by hydrodynamics, monsoonal forcing, and estuarine inputs, exhibiting clear seasonal patterns. For instance, simulations in Chinese coastal waters reveal that floating microplastics achieve the longest transport distances in summer, while remaining predominantly nearshore during autumn and winter (Liu et al., 2023). In the Adriatic Sea, the coastline serves as the primary microplastic deposition sink, with surface concentrations exhibiting significant seasonal variability (Liubartseva et al., 2016). Additional research highlights that microplastics discharged from coastal cities primarily accumulate along shorelines and adjacent islands, with estuarine regions experiencing particularly severe contamination and elevated abundances during the rainy season (Jiang et al., 2022; Li et al., 2023). These findings are consistent with the simulated nearshore high-concentration zones and source-region input patterns in the Beibu Gulf, providing strong support for the proposed seasonal dispersion mechanisms of microplastics in the region.

### 3.3 Effect of vertical velocity on microplastic transport

**FIGURE 6:**
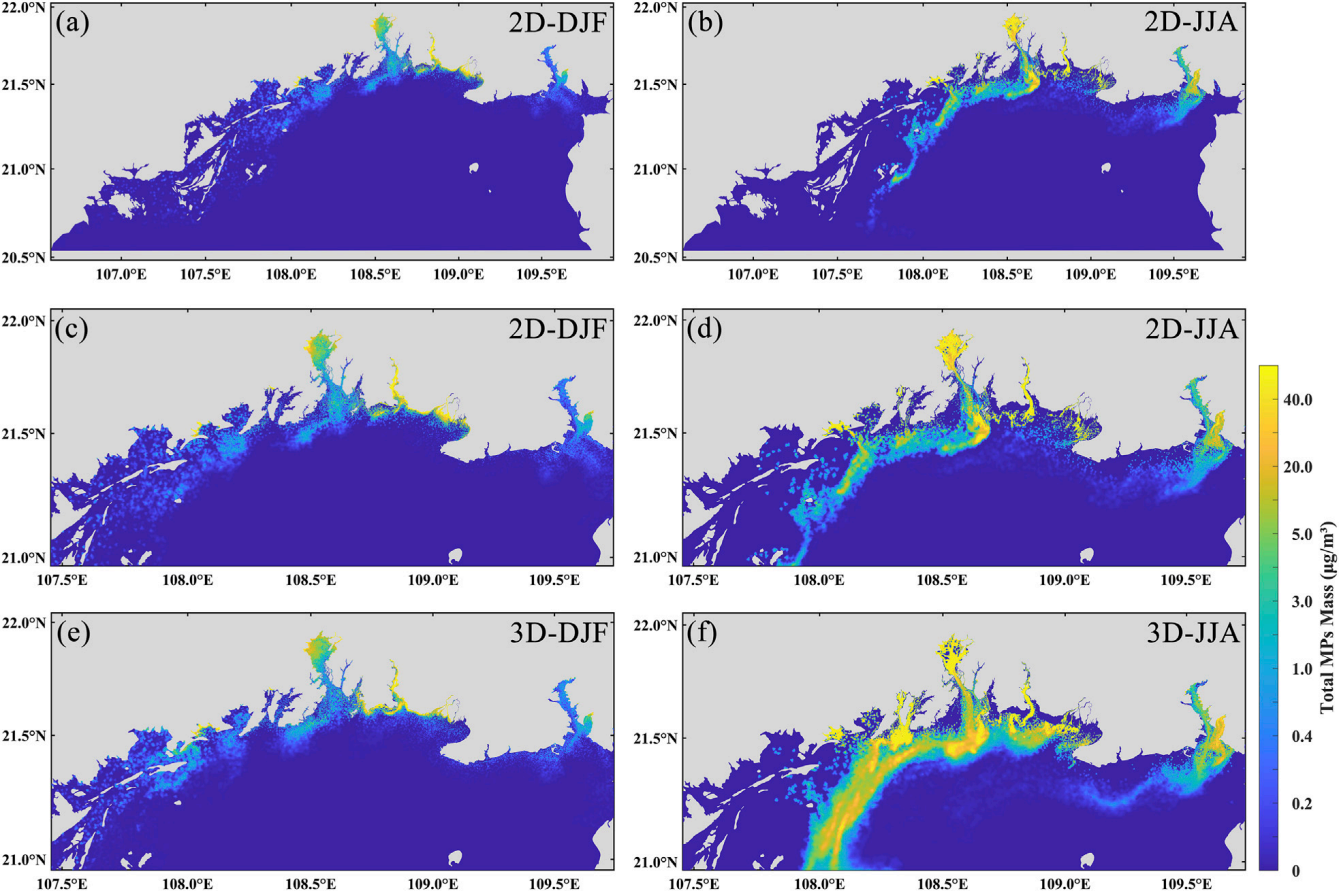
Spatial distribution of microplastics in the case of vertical flow velocity (3D) versus no vertical flow velocity (2D). **(a,b)** shows the two-season distribution of the 2D model; **(c,d)** shows the two-season localized distribution of the 2D model; **(e,f)** shows the two-season localized distribution of the 3D model.

To investigate the influence of vertical velocity on the transport and distribution of microplastics, this study selected two representative seasons for analysis: winter, characterized by the minimum dispersion range, and summer, characterized by the maximum dispersion range. To further examine the role of vertical velocity, we used a two-dimensional model to simulate microplastic transport under conditions without vertical flow. The parameter settings of the two-dimensional model are consistent with those of the three-dimensional model to ensure comparability, thereby allowing a clear assessment of the regulatory effect of vertical velocity on microplastic transport. Calculations indicate that the vertical velocity in the study area ranges from −0.0434 m/s to 0.0236 m/s in summer, with a maximum absolute value of 0.0434 m/s, and from −0.0336 m/s to 0.0275 m/s in winter, with a maximum absolute value of 0.0336 m/s. In terms of mean vertical velocity, the range is −0.00036 m/s to 0.00022 m/s in summer and −0.00022 m/s to 0.00029 m/s in winter. Overall, vertical motions are more intense in summer, indicating more active vertical water exchange, which facilitates the vertical resuspension and mixing of microplastics. The simulated distribution patterns (Figure 6) reveal distinct spatial variations and concentration differences in microplastics under different conditions. In winter (Figures 6a,c,e), microplastics are mainly concentrated in Fangchenggang Bay, Qinzhou Bay, Lianzhou Bay, Tieshangang Bay, and adjacent nearshore areas. In the absence of vertical velocity, the overall dispersal area is reduced, with the plume-covered area and maximum offshore transport distance decreasing to 1,359 km^2^ and 170 km, respectively—representing reductions of approximately 32.2% and 6.3%. In summer, the spatial distribution of microplastics has shifted significantly westward, with high concentrations no longer concentrated in the central part of the sea (Figures 6b,d,f). When vertical velocity is excluded, the plume-covered area and maximum transport distance decrease to 1,407 km^2^ and 177 km, respectively—reductions of approximately 71.7% and 4.2%.

These results suggest that the vertical velocity has a relatively minor influence on the transport direction and distance of microplastics. Although it does not play a dominant role in controlling the transport direction, the vertical velocity significantly affects the dispersion and concentration distribution of microplastics. Moreover, a consistent seasonal-independent pattern is observed: vertical velocity tends to promote the dispersion and spreading of microplastics. During summer, enhanced southwesterly monsoon winds and riverine discharge intensify vertical circulation, bringing deeper microplastics to the surface layer and facilitating their wider dispersal. In addition, the prevailing circulation patterns and coastal upwelling processes in summer further contribute to offshore microplastic transport (Gao et al., 2015; Pang et al., 2025). In winter, strong northeasterly winds can induce intense vertical mixing (Gao et al., 2017), which drives surface microplastics in nearshore areas downward and offshore, resulting in lower surface concentrations compared to conditions without vertical flow.

### 3.4 Impact of storm surges on microplastic transport

**FIGURE 7:**
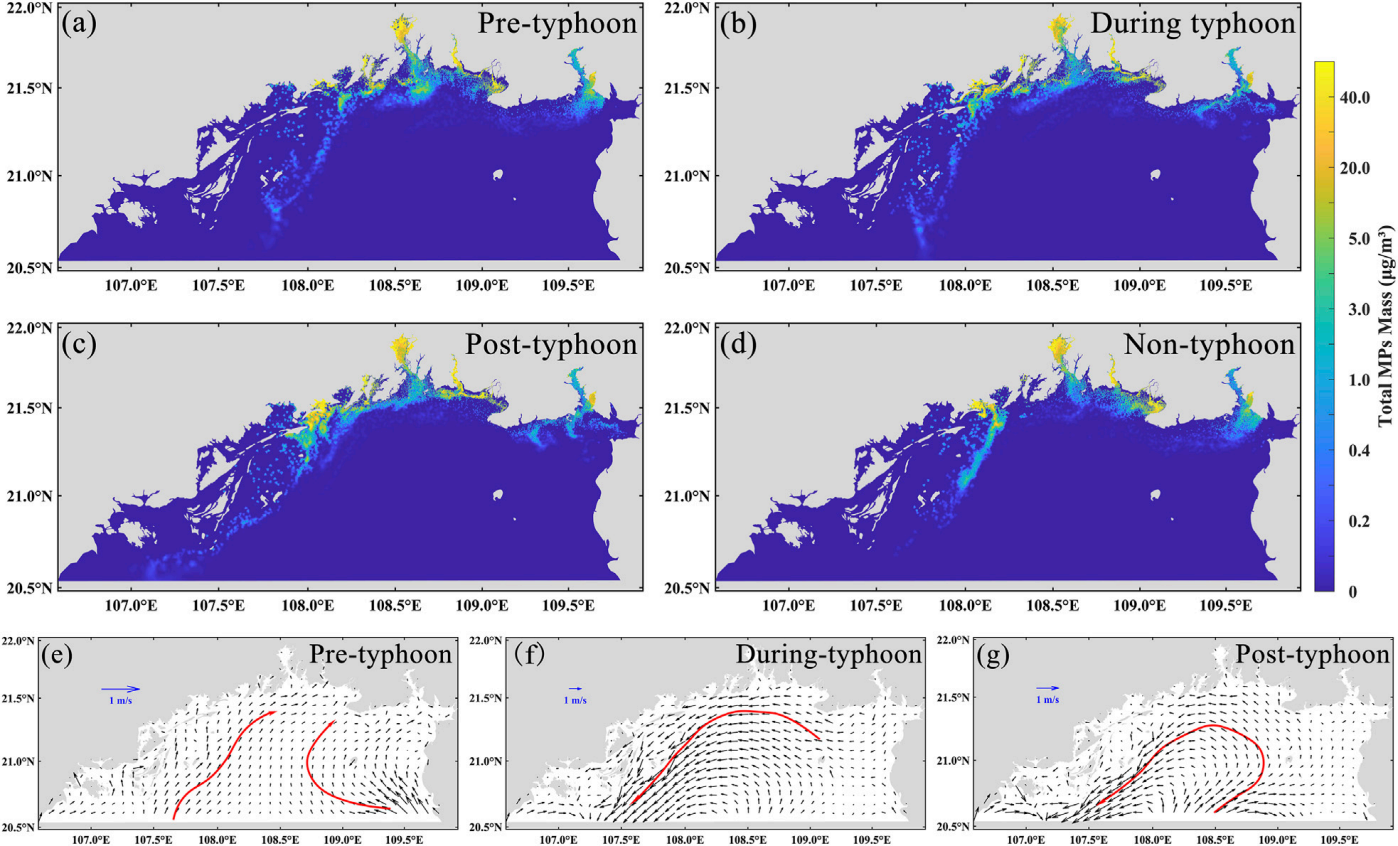
Simulated spatial distribution of microplastics in Beibu Gulf during Typhoon Yagi in 2024. **(a-d)** are the distributions of microplastics before, during, after, and without the typhoon, respectively; **(e–g)** are the flow fields in the Beibu Gulf before and after the typhoon; the red arrows show the main direction of the flow field at that moment.

We further investigated another key factor influencing microplastic dispersion in the Beibu Gulf—storm surges. Based on the simulation results of Typhoon Yagi, we systematically analyzed the spatial distribution characteristics and evolution mechanisms of microplastics before, during, and after the typhoon, as well as under non-typhoon conditions (Figure 7). Prior to the typhoon event, microplastics were mainly concentrated in nearshore areas such as river estuaries and harbors, which are characterized by strong land-based inputs. A distinct nearshore high-concentration zone and a clear land–sea concentration gradient were observed (Figure 7a). During the typhoon event, strong wind stress, wave-induced turbulence, and alterations in large-scale circulation markedly modified microplastic transport and dispersion, leading to accumulation along the coastline and the formation of filamentous or patchy high-concentration bands (Figure 7b). Following the typhoon, the transport distance of microplastics increased substantially, with elevated concentrations observed west of the Beilun River and along the Vietnamese coast, and an overall transport pattern indicating southwestward dispersal along the coastline (Figure 7c). Compared to the distribution under normal hydrodynamic conditions (Figure 7d), the typhoon significantly enhanced the spatial redistribution of microplastics, highlighting its strong facilitative effect on offshore transport. Under typical hydrodynamic conditions, the limited circulation in the Beibu Gulf constrains offshore microplastic transport. The typhoon-induced storm surge disrupted this limitation, substantially intensifying hydrodynamic conditions: prior to the typhoon, tidal forces dominated circulation with maximum velocities of approximately 0.56 m/s, whereas during the typhoon, storm-driven flow velocities reached up to 2.8 m/s. This pronounced increase in flow speed accelerated the dispersal of microplastics toward offshore waters, extending their transport distance by approximately 68 km compared to non-storm conditions, thereby significantly altering the spatial distribution patterns of microplastics in the Beibu Gulf.

Further analysis reveals that the typhoon event exerted a significant influence on the hydrodynamic characteristics of the Beibu Gulf. Prior to the typhoon, circulation was primarily tide-driven, with flow directions varying periodically in response to the tidal cycle. During the mid and late stages of the typhoon, however, storm-induced intense circulation generated a pronounced counterclockwise vortex within the gulf (Figures 7f,g). This vortex not only altered transport pathways but also had a substantial impact on microplastic distribution: a portion of the microplastics accumulated along the coastline under the vortex’s influence, while another portion was advected offshore, exhibiting clear spatial redistribution. The dominant mechanisms driving microplastic transport during the storm surge comprised variations in surface flow velocity, wind-stress–induced convection and vertical mixing (Nel et al., 2018; Prevenios et al., 2018), vortex circulation structures, and the cumulative effects of storm surge dynamics. The formation of offshore high-concentration zones after the typhoon is largely attributed to the counterclockwise mesoscale eddies generated during the storm event. Following the typhoon, nearshore circulation exhibited a reversal in flow direction. On one hand, this reversal transported part of the microplastics back toward the coast; on the other, it facilitated the formation of secondary accumulation zones in semi-enclosed or leeward areas. In addition, intense storm surges can induce the resuspension of microplastics embedded in marine sediments, introducing them back into the surface water layer. This process expands the spatial distribution of microplastics while enhancing their suspension and persistence in the water column (Lo et al., 2020), thereby exacerbating the overall environmental load. Extreme weather events such as typhoons not only alter the short-term distribution patterns of microplastics, but also have the potential to reshape their migration pathways among the water column, seabed sediments, and coastal zones, thereby significantly affecting the regional fate of microplastics.

## 4 Conclusion

In this study, a three-dimensional numerical model coupled with a Lagrangian particle tracking module was employed to simulate the migration and dispersion of microplastics in the Beibu Gulf. The model’s reliability was assessed through comparison with observed drifter trajectories. The simulation results revealed the seasonal characteristics of riverine microplastic transport, as well as the effects of vertical velocity and storm surges. The main conclusions are as follows:

1. Microplastic transport exhibits pronounced seasonal variability. Driven by the counterclockwise coastal current, microplastics predominantly disperse southwestward throughout all four seasons. In spring, autumn, and winter, pollution plumes are concentrated near estuarine zones and along the Vietnamese coast. In summer, under the influence of the southwest monsoon, transport pathways shift toward the central gulf, substantially expanding the affected region and generating high-concentration zones.
2. Vertical velocity has a significant impact on the spatial distribution of microplastics. Although its influence on transport distance is limited, vertical flow markedly alters the coverage area of the pollution plume and the location of high-concentration zones.
3. Using Typhoon Yagi as an example, the storm surge rapidly altered the spatial distribution of microplastics in the Beibu Gulf. Prior to the typhoon, microplastics were mainly concentrated in nearshore waters; during the event, strong wind stress and circulation forced their accumulation along the coastline while driving dispersion offshore. After the typhoon, transport distances increased, with an overall southwestward dispersal pattern. Compared to non-typhoon conditions, the storm surge enhanced the redistribution capacity of microplastics, promoted offshore dispersion, and reshaped their transport pathways within the gulf through vortex-driven circulation.

However, this study still has certain limitations and does not fully capture the distribution characteristics of microplastics in real marine environments. The source input settings considered only riverine inputs, and the use of single-point microplastic observation data cannot accurately represent the actual transport flux from rivers, thus affecting the accuracy of the input data. In addition, the parameterization of processes such as microplastic settling, degradation, and resuspension in the Lagrangian particle tracking model remains simplified, limiting the representation of the complex biogeochemical behavior of microplastics. These limitations suggest that future research should incorporate multi-source observational data to improve the estimation of input fluxes and further introduce dynamic processes involving interactions between microplastics and environmental factors, in order to enhance the accuracy and applicability of simulation results.

## Data Availability

The original contributions presented in the study are included in the article/Supplementary Material, further inquiries can be directed to the corresponding authors.
