# Supplementary material for: Simulation of microplastic transport and dispersion based on a three-dimensional hydrodynamic particle-tracking model in the Beibu Gulf
Source: Front Toxicol. 2025 Dec 4;7:1676823. doi: 10.3389/ftox.2025.1676823 (PMC12712604; doi:10.3389/ftox.2025.1676823)
Supplement: Supplementary file 1 [file DataSheet1.pdf]

The following are the partial verification results of the model:

Table 1 Observation Information and Parameters.

Station	Lon (°E)	Lat (°N)	Investigation	Observation Period
T1	109.0833	21.4833	Tide	December 15, 2024 - December 20, 2024
T2	109.1167	21.0167	Tide	
T3	109.5667	21.5833	Tide	
T4	109.7667	21.2667	Tide	
T5	108.5500	21.7500	Tide	
T6	108.3333	21.6000	Tide	
T7	108.2333	21.5000	Tide	
S1	109.1983	21.0941	Hydrodynamics	December 15, 2024 - December 16, 2024
S2	109.1926	20.9452	Hydrodynamics	
S3	109.4170	21.2684	Hydrodynamics	
S4	109.4154	20.9310	Hydrodynamics	
S5	109.1938	21.2637	Hydrodynamics	
S6	109.4180	21.0945	Hydrodynamics	

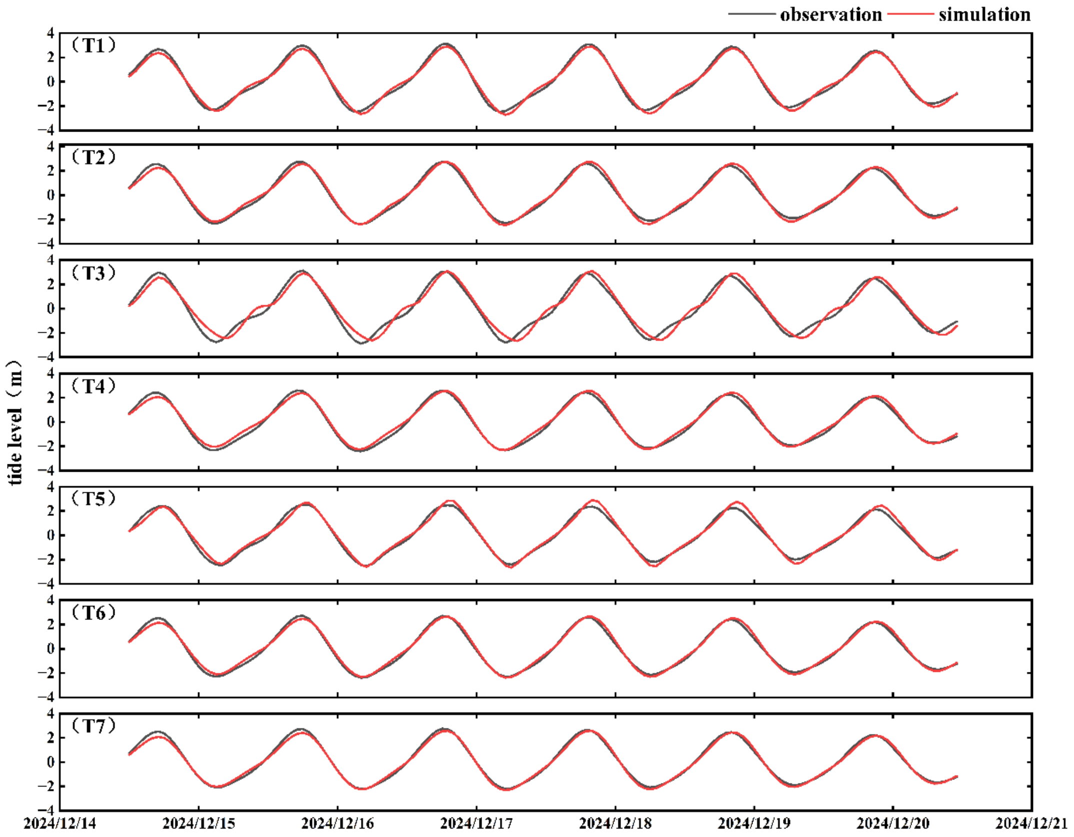

Figure 1 Tide Level Verification Results

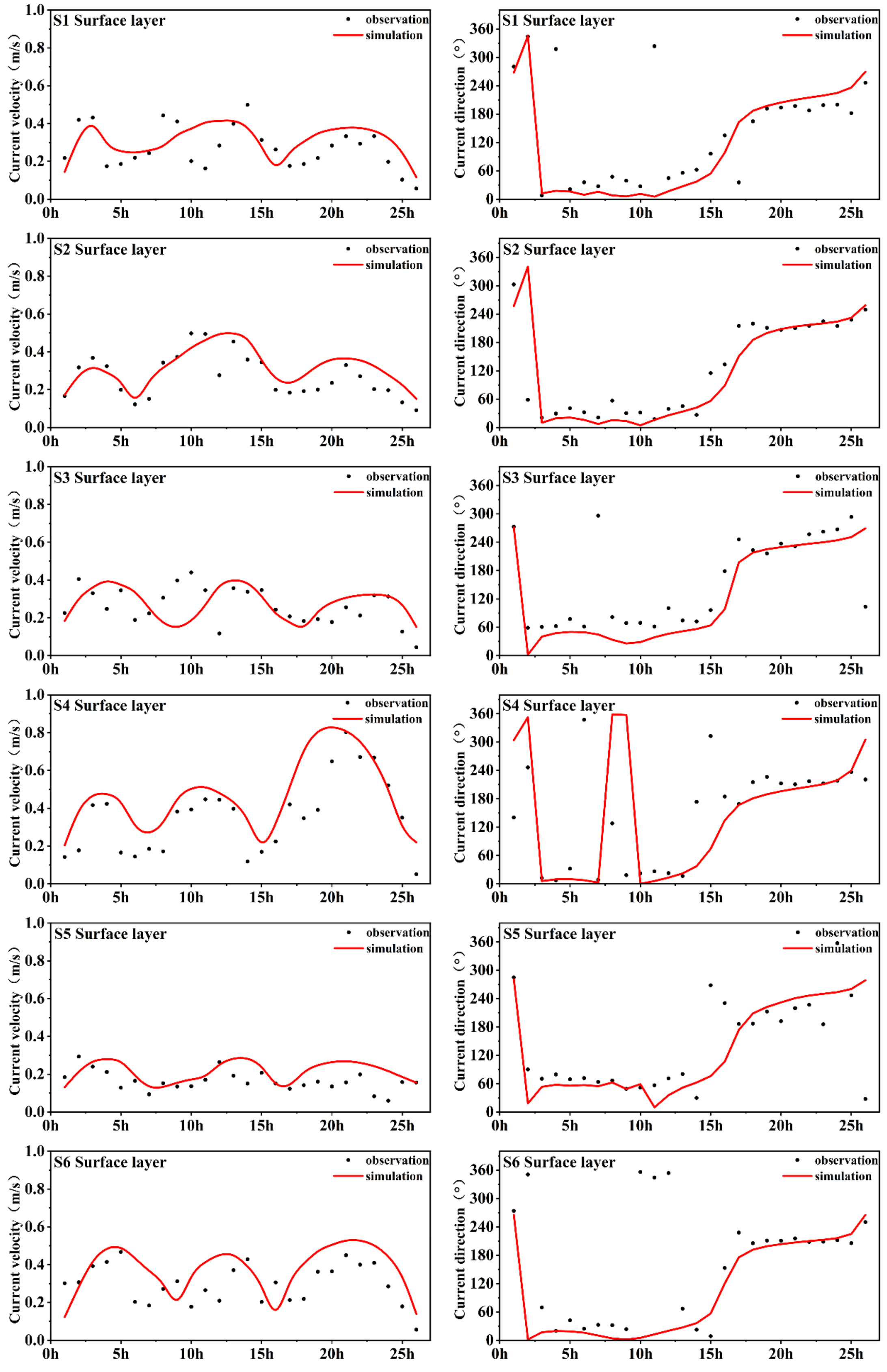

Figure 2 Surface Current Velocity and Direction Verification Results for Stations S1-S6

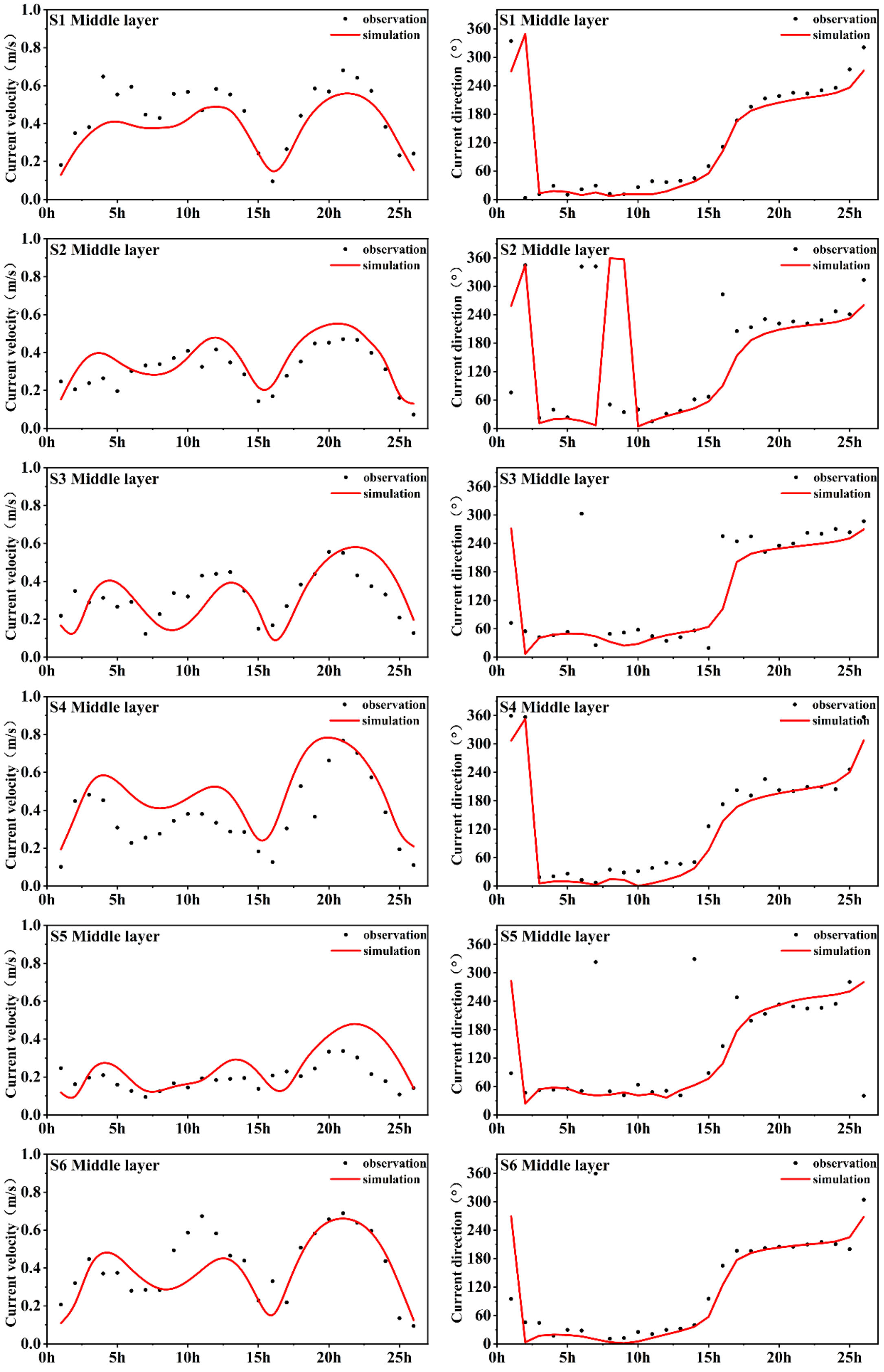

Figure 3 Mid-layer Current Velocity and Direction Verification Results for Stations S1-S6

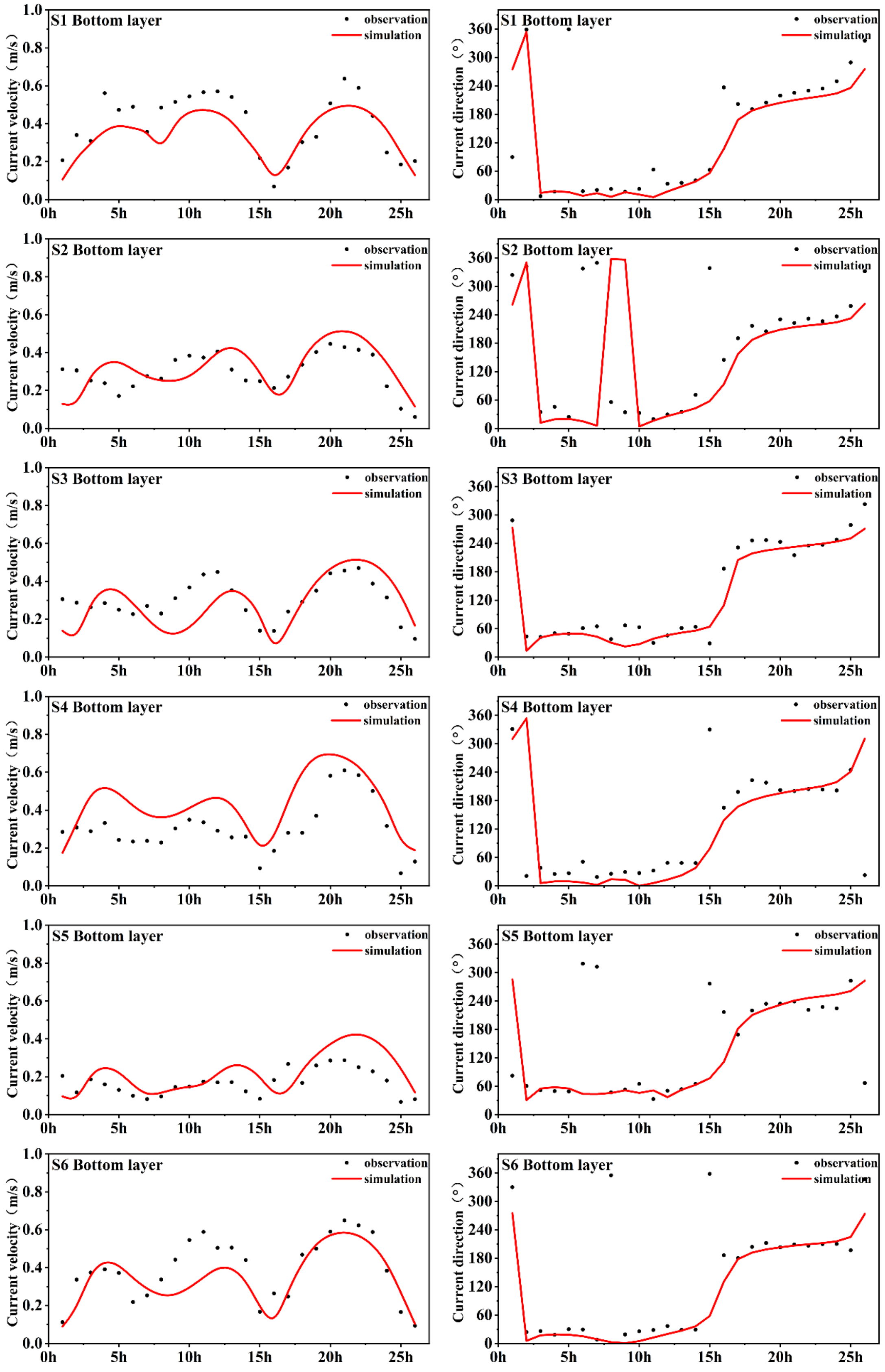

Figure 4 Bottom-layer Current Velocity and Direction Verification Results for Stations S1-S6
